# Toward a Species Search Engine: KISSE Offers a Rigorous Statistical Framework for Bone Collagen Tandem Mass Spectrometry Data

**DOI:** 10.1002/advs.202503963

**Published:** 2025-08-11

**Authors:** Hassan Gharibi, Amir Ata Saei, Alexey L. Chernobrovkin, Susanna L. Lundstrom, Hezheng Lyu, Zhaowei Meng, Akos Vegvari, Massimilliano Gaetani, Roman A. Zubarev

**Affiliations:** ^1^ Division of Chemistry I Department of Medical Biochemistry and Biophysics Karolinska Institutet Stockholm 17177 Sweden; ^2^ Chemical Proteomics Swedish National Infrastructure for Biological Mass Spectrometry (BioMS) Stockholm 171 77 Sweden; ^3^ Chemical Proteomics Unit SciLifeLab Stockholm 171 77 Sweden; ^4^ Department of Microbiology Tumor and Cell Biology Karolinska Institutet Stockholm 171 77 Sweden; ^5^ Pelago Bioscience Solna 171 65 Sweden; ^6^ Department of Pharmacological & Technological Chemistry I.M. Sechenov First Moscow State Medical University Moscow 119146 Russia; ^7^ Department of Pharmaceutical and Toxicological Chemistry Medical Institute RUDN University 6 Miklukho‐Maklaya St Moscow 117198 Russia

**Keywords:** collagen sequences, LC‐MS/MS, proteomics, species Identification

## Abstract

DNA and bone collagen are two key sources of resilient molecular markers used to identify species from their remains. Collagen is more stable than DNA, and thus it is preferred for ancient and degraded samples. Current mass spectrometry‐based collagen sequencing approaches are empirical and lack a rigorous statistical framework. Based on the well‐developed approaches to protein identification in shotgun proteomics, a first approximation of the species search engine (SSE) is introduced. SSE named KISSE is based on a species‐specific library of collagenous peptides that uses both peptide sequences and their relative abundances. The developed statistical model can identify the species and the probability of correct identification, as well as determine the likelihood of the analyzed species not being in the library. The advantages and limitations of the proposed approach, and the possibility of extending it to other tissues is discussed.

## Graphical abstract

The Species Search Engine (KISSE) is a novel statistical approach for identifying species from collagen peptides, using a curated library of sequences and their relative abundances derived from shotgun proteomics.

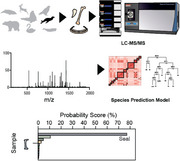

## 1 Introduction

The identification of animal species by bone fragments, including fossilized samples, is of utmost importance in several scientific disciplines, such as paleontology, archaeology, and forensics. This information helps scientists better understand the evolution, behavior, and interaction of the species with the environment.^[^ ^1^ ^]^ For example, in paleontology, protein analysis from fossil bones has been used to identify extinct species such as mammoths and ground sloths, shedding light on ancient biodiversity and environmental conditions.^[^ ^1^ ^]^ In forensics, the identification of bone samples can aid in criminal investigations and the documentation of human remains. For instance, collagen fingerprinting has been applied to distinguish between human and non‐human bones in complex forensic contexts.^[^ ^2^ ^]^ Additionally, in archaeology, species identification has helped determine the origin of fragmented hominin bones from the Denisova Cave, allowing researchers to link them to Denisovans and Neanderthals and providing new insights into human ancestry and evolution.^[^ ^3^ ^]^ To a large extent, the ability to accurately identify animal species from pieces of bones can advance our understanding of the evolution of the natural world and human history.^[^ ^3^ ^]^

In a wide range of disciplines, including forensic anthropology,^[^ ^4^, ^5^ ^]^ archaeology,^[^ ^6^ ^]^ and evolutionary biology,^[^ ^7^ ^]^ Morphological analysis is a foundational method for the identification of biological specimens. Traditionally, experts assess and compare physical features such as bone and dental structures, particularly when matching antemortem and postmortem data in forensic contexts.^[^ ^4^, ^5^ ^]^ These assessments are often qualitative and rely heavily on visual comparisons, making them valuable as an initial step in identification.^[^ ^4^ ^]^ However, visual and morphological approaches alone can sometimes be insufficient, especially when distinguishing between closely related species whose skeletal structures are highly similar.^[^ ^8^ ^]^ In such cases, molecular identification methods can be useful. By analyzing species‐specific variations in DNA or proteins, molecular tools enable more precise taxonomic classification, independent of morphological features.^[^ ^8^ ^]^

While genetic analysis of DNA extracted from the bone provides the most definite species identification, collagen, a protein found in connective tissues such as bones and cartilage, has been found to be more resistant to degradation and easier to extract from fossils and ancient remains than DNA.^[^ ^9^ ^]^This is because collagen is much more abundant in the bone than DNA, and its triple protein helix is more stable than the DNA double helix against various environmental stressors, such as heat, moisture, and exposure to ultraviolet radiation.^[^ ^10^ ^]^ Also, collagen sequences in different tissues and organs can vary, unlike DNA, and thus offer the possibility to identify, at least to some degree, not only the species but also the tissue.^[^ ^11^, ^12^ ^]^ In addition, racemization and deamidation of amino acid residues in collagen can provide valuable information about the age and conditions of bone preservation, which can help researchers better understand the context of the remains.^[^ ^13^ ^]^ Therefore, in some areas of science, such as paleoproteomics,^[^ ^14^, ^15^ ^]^ evolutionary biology,^[^ ^16^ ^]^ and archaeology,^[^ ^17^ ^]^ collagen analysis has gained more popularity than DNA sequencing.

At first glance, species identification by collagen analysis appears straightforward: collagen can be readily extracted from bone, enzymatically digested (e.g., with trypsin), and analyzed via mass spectrometry (MS).^[^ ^18^ ^]^ The earliest proteomics approach, peptide mass fingerprinting (PMF), was developed to match the molecular masses of these peptides to known sequences in a database.^[^ ^17^, ^19^, ^20^ ^]^ Although PMF proved useful, especially in analyzing ancient or degraded samples,^[^ ^10^ ^]^ it provides limited resolution, as many collagen type I peptides are conserved across closely related species, hindering precise taxonomic identification.^[^ ^21^ ^]^ Moreover, tools like MASCOT, widely used in proteomics, are not optimized for species‐level classification, often requiring manual identification of a few diagnostic peptides.^[^ ^22^ ^]^ This reliance on expert interpretation is labor‐intensive and compromises scalability, reproducibility, and objectivity.^[^ ^22^ ^]^ To address this, tools such as PAMPA were developed to standardize and automate peptide marker‐based classification within the PMF framework.^[^ ^22^ ^]^

Nonetheless, over time, PMF has shown significant limitations and, after extensive attempts to improve it, has been largely abandoned in mainstream proteomics due to its lack of statistical robustness,^[^ ^21^ ^]^ peptide mapping specificity and sensitivity,^[^ ^23^, ^24^ ^]^ and the capability to process complex proteomics samples.^[^ ^25^ ^]^ This prompted a shift toward tandem mass spectrometry (MS/MS), which fragments peptide ions in the gas phase to yield sequence‐specific fragment spectra and improved identification confidence.^[^ ^26^, ^27^ ^]^ MS/MS enables not only more accurate species assignments but also phylogenetic insights across taxa.^[^ ^28^ ^]^ However, even with MS/MS, challenges remain: protein identification based on a single peptide match, a “one‐hit wonder”, is generally regarded as unreliable.^[^ ^29^ ^]^

To mitigate these issues, recent MS/MS‐based workflows have introduced more robust and automated solutions. For example, the Species by Proteome Investigation (SPIN) approach integrates fast, automated sample prep, data‐independent acquisition (DIA), and an FDR‐controlled species inference algorithm to achieve high‐throughput and high‐resolution identification of both collagenous and non‐collagenous proteins—even in severely degraded samples.^[^ ^8^ ^]^ Similarly, ClassiCOL uses a curated collagen database (CollagenDB) and two novel algorithms—isoBLAST and ClassiCOL—to handle isobaric peptide variants and classify species by systematically rejecting improbable taxonomic paths rather than relying on a fixed set of diagnostic peptides.^[^ ^30^ ^]^ This approach improves resolution and allows detection of mixed samples or taxa not fully represented in current databases.^[^ ^30^ ^]^

Understanding how species are identified via MS/MS requires a brief review of standard bottom‐up proteomics workflows. Fragment spectra are searched against a protein sequence database (plus a decoy database, often composed of reversed sequences) using scoring algorithms that simulate expected fragmentations and calculate the probability of a match.^[^ ^31^, ^32^, ^33^ ^]^ Confidence is quantified using E‐values and further refined by estimating the false discovery rate (FDR), based on hits to the decoy database.^[^ ^34^, ^35^ ^]^ To assign peptides to proteins, a common principle is applied, only proteins with at least two unique peptide identifications are typically accepted to ensure reliability.^[^ ^36^, ^37^ ^]^

The above procedure is demonstrably complex, but this complexity serves an important purpose – proteomics data are far more trustworthy today than in previous decades. However, still, little of this complexity is implemented in the approaches used for species identification from bone collagen, even though the number of mammal species known (≈6,400 species^[^ ^38^ ^]^) is of the same order of magnitude as, e.g., the size of the yeast proteome (≈6000 proteins^[^ ^39^ ^]^). There are also bones of ≈10,000 bird species,^[^ ^40^ ^]^ and ≈32,000 species of fish,^[^ ^41^ ^]^ while in archaeological contexts, faunal assemblages often comprise a mixture of taxa from various classes.^[^ ^42^, ^43^, ^44^ ^]^ Moreover, protein sequences in a proteome are quite diverse, while most collagen proteins from even distant species are highly homologous.^[^ ^45^ ^]^ This calls for an even more vigorous procedure for species identification by bone collagen analysis than the protein identification routine in regular proteomics. We call this hypothetical procedure a “species search engine” or SSE, similar to protein search engines in shotgun proteomics.

Developing a satisfactory SSE will require the combined efforts of many bioinformatics groups and will likely take several years. Here we present the first draft of an SSE, named KISSE, based on bone collagen data from 8 species (38 individuals in total): *Halichoerus grypus* (grey seal), *Phoca vitulina* (harbor seal, herein referred to as “seal”), *Ziphius cavirostris* (Cuvier's beaked whale, “cuvier” for short), *Physeter macrocephalus* (sperm whale), *Eschrichtius robustus* (grey whale), *Falco peregrinus* (peregrine falcon, “falcon”), *Cygnus cygnus* (whooper swan, “swan”) and the extinct *Hydrodamalis gigas* (Steller's sea cow, “sea cow”). The approach taken does not aim to establish a definitive path for the future but rather focuses on making the most efficient use of current resources. KISSE predicts the most likely species, the next likely species, and estimates the statistical certainty of the results. When the latter value drops below a certain threshold, an indication is obtained that the analyzed species may not be in the database. When a new species is added to KISSE, the scoring and the probability systems need to be recalculated.

**Figure 1:**
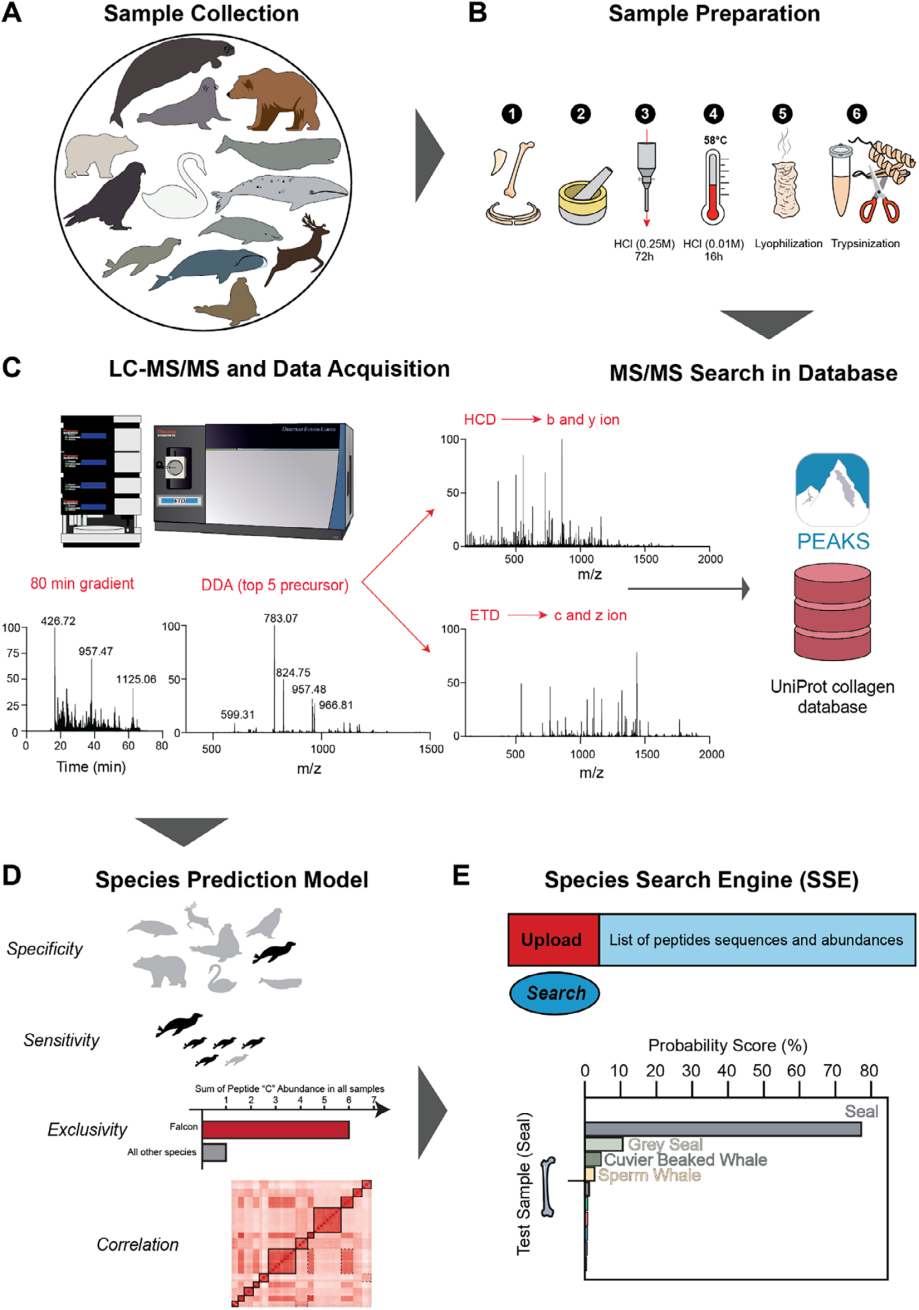
Workflow of developing KISSE – the first Species Search Engine. A), Sample collection. B), Collagen extraction from bone pieces: 1. Washing the bone or tooth with water, 2. Grinding the bone or tooth pieces into powder, 3. Demineralization of bone powder with HCl to remove calcium from the collagen matrix, 4. Gelatinization with HCl at 58 °C to denature collagen followed by ultra‐filtration through a 30 kDa cutoff filter, 5. Lyophilization of denatured collagen solution using a freeze‐dryer, 6. Tryptic digestion of collagen. C), LC‐MS/MS analysis of tryptic peptides, data‐dependent acquisition (DDA) with HCD and ETD MS/MS, and usage of PEAKS Studio to perform the search of the.raw files against UniProt collagen database and quantification based on the extracted ion chromatogram of the molecular ions. D), Building the Species Predicting model using Specificity, Sensitivity, Exclusivity as parameters for peptide selection, and correlation of peptide abundances as an augmenting factor. E), User Interface, and the output of the SSE online tool providing the most probable species and the estimated probability score of correct identification.

Working on the KISSE development, it became necessary to analyze bones from several individuals of the same species for including the species in the database, as the provenance of a bone sample is not always known with absolute certainty. As shown in **Figure** **1**A, our initial species collection comprised 5 more species: *Ursus maritimus* (polar bear), *Ursus arctos* (brown bear), *Mirounga leonine* (southern elephant seal), *Balaena mysticetus* (bowhead whale), and *Rangifer tarandus* (reindeer). However, these were later excluded due to the lack of a sufficient number of individuals per species (at least three). Bone and tooth samples underwent collagen extraction following the Brown protocol^[^ ^46^ ^]^ (Figure 1B). After collagen extraction, digestion, and desalting procedures, label‐free peptides were separated by liquid chromatography and analyzed on a high‐resolution mass spectrometer equipped with both Higher Collision Dissociation (HCD) and electron‐transfer dissociation (ETD) MS/MS. Proteomics data were recorded in a Data Dependent Acquisition (DDA) mode, and the top 5 precursors in each survey mass spectrum were selected for both HCD and ETD MS/MS. This enabled reliable database identification^[^ ^47^ ^]^ as well as accurate *de novo* sequencing,^[^ ^48^ ^]^ if needed. The MS/MS data were then searched against UniProt/SwissProt reviewed collagenous proteins as well as any protein in the extracellular matrix in the PEAKS studio software^[^ ^49^ ^]^ (Figure 1C).

To build a Species Identification and Prediction by Mass Spectrometry (SIP‐MS) model, a subset of peptides was selected based on their specificity, sensitivity, and exclusivity (for details see Experimental Procedures), as well as on the correlation of peptide abundances in individual samples (Figure 1D). The SIP‐MS model contains 1) a random forest classification that uses the top N (N = 11) most species‐specific peptides to predict the taxonomy of a given sample, and 2) a correlation matrix based on the peptide abundances to augment the random forest prediction results and provide the final species identification.

The obtained model for species prediction was then tested based on the out‐of‐bag (OOB) evaluation technique. OOB is a built‐in way to assess model performance without needing a separate test set. The OOB estimate leverages the fact that each decision tree in the random forest is trained on a bootstrapped sample of the data, meaning that some observations (usually about one‐third) are left out of each sample. These out‐of‐sample observations are then used to evaluate model performance for each tree, and this forms the OOB estimate. To determine the probability score threshold for reliable identification, the model was further tested on known species that were not in the library. The tested model was placed on the web as an R Shiny application (https://kisse.serve.scilifelab.se/app/kisse) with a graphical user interface that provides the most probable species and the estimated probability of correct identification when the list of peptides and their abundance is uploaded. The external users can also extend the library of specific peptides, adding new species or modifying the existing database (See Experimental Procedure).

## 2 Results

In the database used for building the prediction model, we included 287 proteins identified with at least two unique peptides in the bones of 8 species. Of these proteins, 168 were various collagen types, such as Collagen alpha‐1(I), Collagen alpha‐1(II), Collagen alpha‐1(III), etc. On average 158 peptides per collagen type were identified. Of the 26,581 peptides identified in total, 2052 peptides were unmodified, 20,775 were containing proline hydroxylation, 6,480 deamidation of asparagine or glutamine, and 1,079 methionine oxidation. Some of the peptides had several modifications. Among all peptides, 9,582 were unique to specific collagen sequences (57 peptides/per collagen type on average). For the species that did not meet the criteria to be included in the training of the prediction model (referred to as “Outside DB”), we identified a total of 248 proteins with at least two unique peptides. In this dataset, 14,483 peptides were included, representing 160 different collagen proteins, with an average of 90 peptides per type. Among these peptides, 917 were unmodified, 11,653 with proline hydroxylation, 3,779 exhibited deamidation of asparagine or glutamine, and 603 carried methionine oxidation. Of these peptides, 4,103 were unique to specific collagens, averaging 26 peptides per type.

### 2.1 Peptide Selection and SIP‐MS Model Building

**Figure 2:**
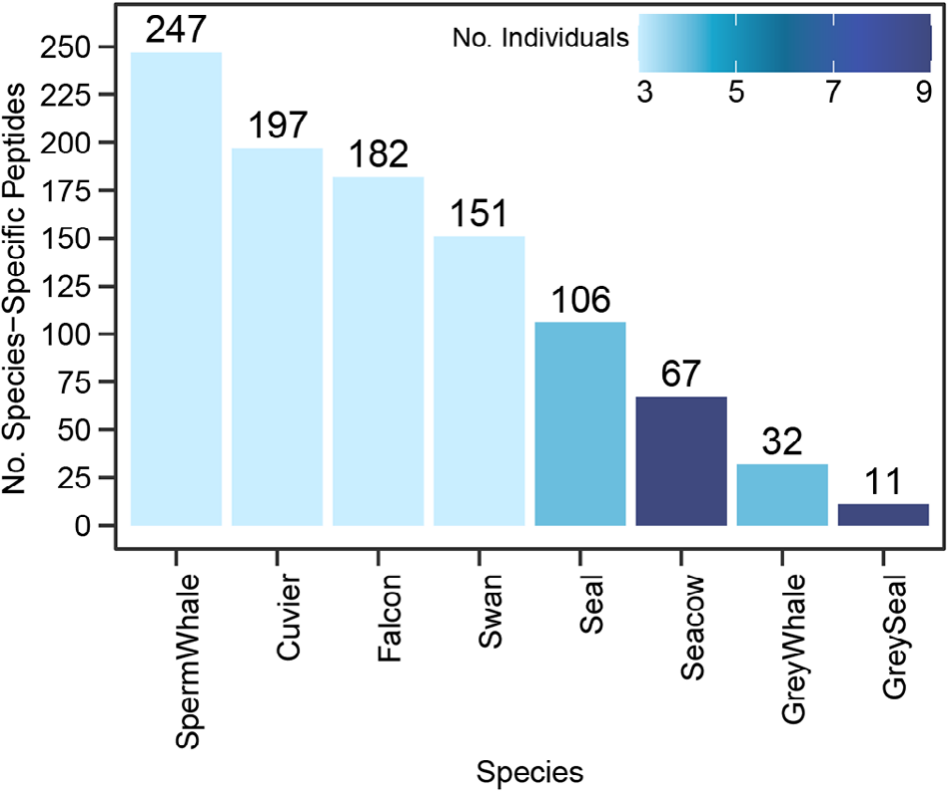
Overview of the Species‐Specific Peptides. Bar plot for the number of Species‐Specific peptides color‐coded by the number of individual samples per species.

For each species in our inventory, we discovered collagenous peptides that were unique (Specificity and Sensitivity value of 1) to a given species (**Figure** **2**). In total, there were 993 species‐specific peptides, while the sperm whale had the highest number of species‐specific peptides, with 247 identified peptides common for 3 different individuals. On the contrary, the grey seal gave the lowest number of species‐specific peptides, with only 11 common peptides found in 9 individuals. One could hypothesize that with more individuals analyzed, fewer common species‐unique peptides will be found, but there was no clear trend between these two numbers. Table S2 (Supporting Information) provides the list of all species‐specific peptides along with their protein accession, peptide sequences, mass, charge state, retention time, and averaged normalized peptide abundance.

**Figure 3:**
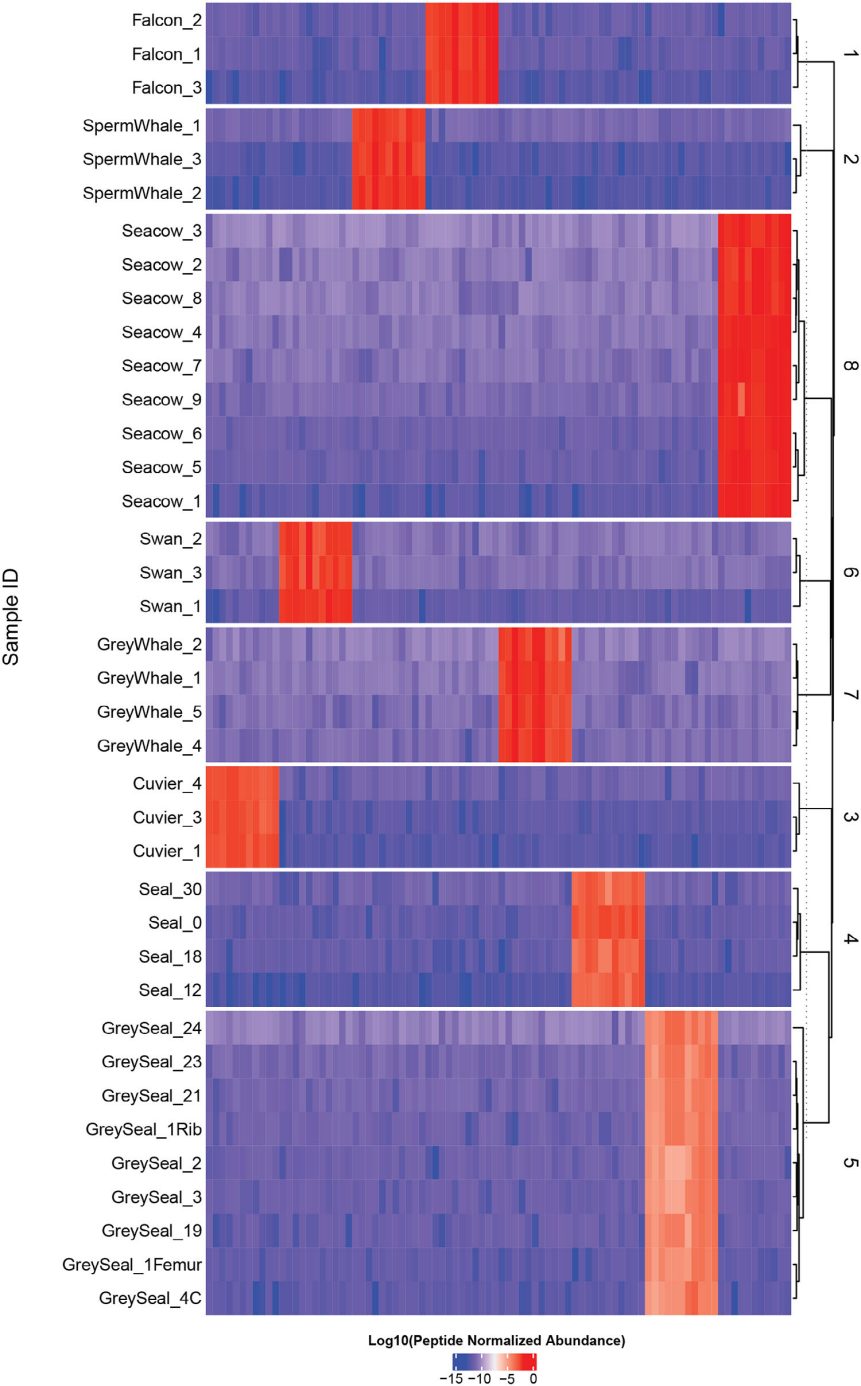
Clustering of 88 species‐specific peptides selected for random forest classification in the SIP‐MS model.

When the peptide is not present in a mass spectrum, the software assigns to its abundance the noise level. To check the dynamic range of such peptide quantification, we created a heatmap of the normalized abundances for the 88 species‐specific peptides selected from the 8 species (**Figure** **3**). Not surprisingly, only clustering of the samples was observed according to the same species, with the dynamic range of abundances stretching for at least 12 orders of magnitude.

**Figure 4:**
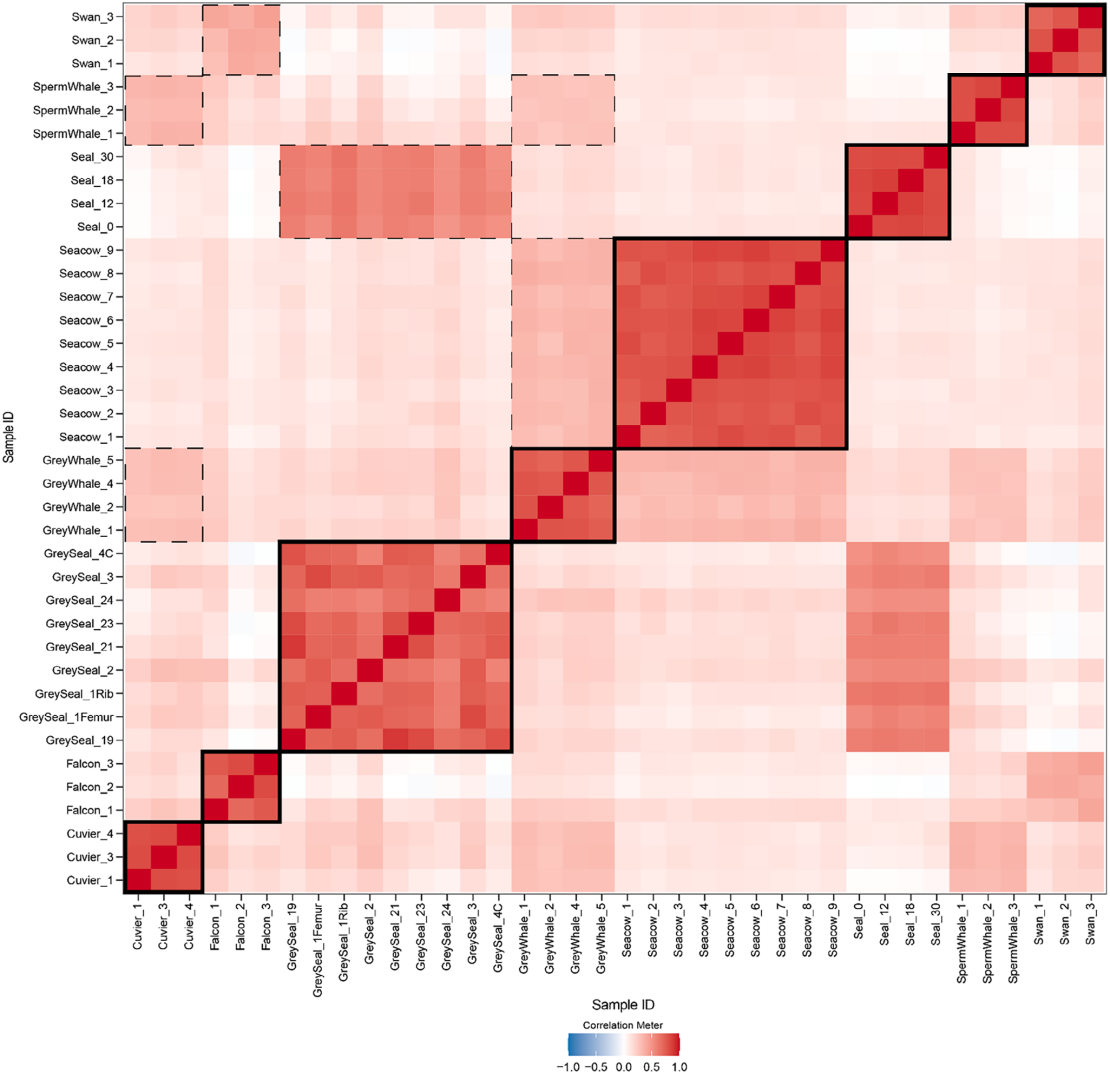
Correlation between the abundances of informative peptides in individual samples. Thick boxes show the clustering for individual samples within each species group, and dashed‐line boxes denote notable inter‐species correlations.

In total, 2,469 other peptides fitted the criteria for informative peptides (see Experimental Procedure). Abundance correlations for these informative peptides in individual samples are shown in **Figure** **4**. Not surprisingly, a much higher correlation was observed for individuals from the same species, but a weaker correlation with related species (grey seal and seal, sperm, grey and Cuvier's whales, falcon and swan, etc.), and the absence of correlation for distant species was also observed.

**Figure 5:**
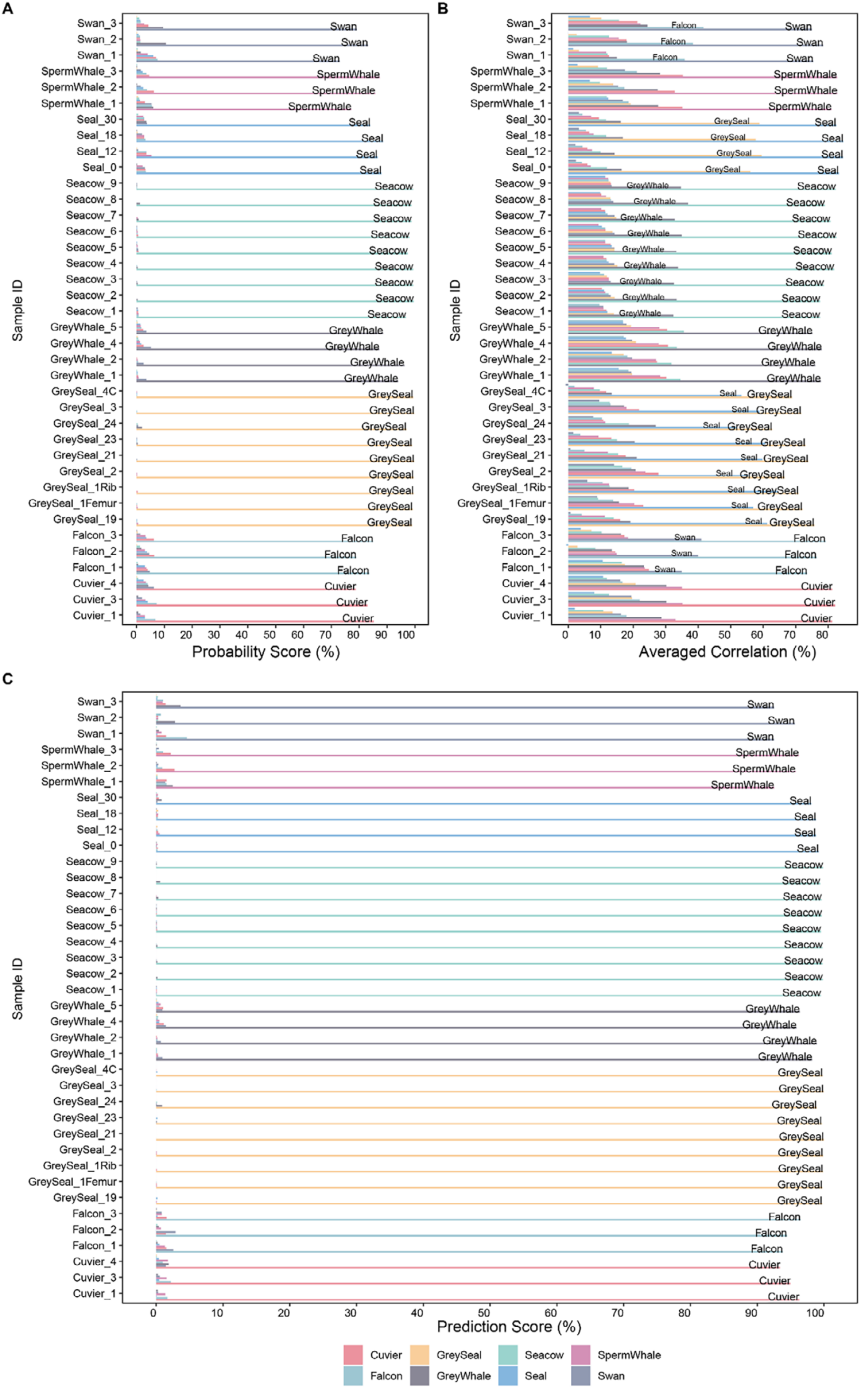
SIP‐MS prediction results for species included in the database. A), Probability scores from the random forest classifier. B), Averaged correlations obtained from informative peptides abundances. C), Final Prediction Score from the SIP‐MS model.

As expected, the random forest classification SIP‐MS model perfectly identified the species included in the training set (**Figure** **5**A). The averaged correlation, which comes from averaging the correlations of the informative peptides of all individuals within each species, is also correctly determining the correct species (Figure 5B). The final prediction scores for each individual sample ranged from 92% to 100% for the correct species identification (Figure 5C). For each species, there was a strong, significant difference (median p‐value < 5×10^−15^) between the prediction of correct ID versus incorrect identification, taking all individuals of every species as a group.

Thereafter we evaluated how the model performs on the five species that were not used for building the SIP‐MS model (“outsideDB” set). For the outsideDB set, peptide abundances were first normalized by the total abundance in each sample, as was done with the training data. To enable comparison with the current database, we retained only the peptides present among the informative peptides in the outside database. We recommend the averaged correlation cut‐off to be set at 0.6, since the least correct correlation species in our dataset (Grey Seal) had an averaged correlation of 0.63. If no species correlates better than the cut‐off averaged correlation with the test sample in the SIP‐MS model, the KISSE assumes that the species is not in the dataset and uses an averaged correlation to provide the identification of the closest species for that sample.

**Figure 6:**
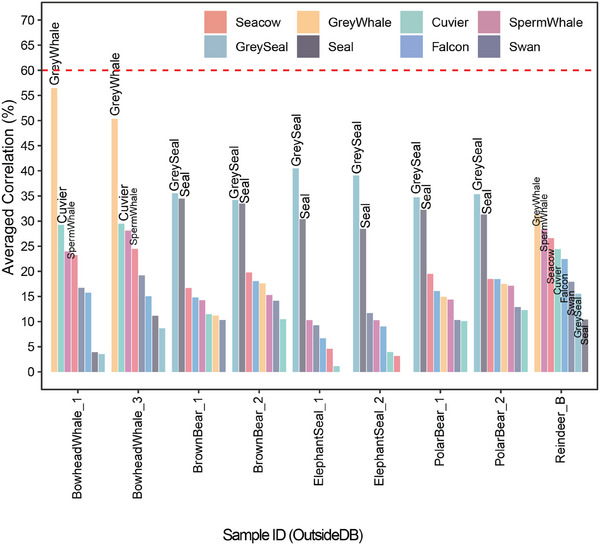
Averaged correlations from the SIP‐MS model for samples of species not included in the database, i.e. outsideDB set. The red dashed line shows the averaged correlation cut‐off used by KISSE to determine if a species exists in our current database.

In **Figure** **6**, it is shown that none of the OutsideDB samples exhibit an averaged correlation surpassing the correlation cutoff on SIP‐MS. Therefore, KISSE concluded that the correct species for these samples is not present in the current database. Instead, KISSE provided the averaged correlation as a means of indicating the most similar species to each sample. In our dataset, the species most similar to bowhead whales is the grey whale, while for brown and polar bears, it is the grey seal, with a median p‐value of 10^−^⁷ for the most similar species. This is consistent with evolutionary relationships: bears and seals are more closely related on the evolutionary tree, both belonging to Carnivora → Caniformia → Arctoidea, and thus the higher similarity scores between them. Reindeer, on the other hand, belongs to the Artiodactyla order, which is more distantly related to any other species currently represented in our database. This greater phylogenetic distance is reflected in the relatively close proximity of the averaged correlation values and a higher p‐value (5 × 10^−^^3^), compared to KISSE's predictions for other OutsideDB samples.

### 2.2 Comparison of Species‐Specific Peptide Identification: MS/MS versus PMF Approaches

To compare the mass sensitivity of species‐specific peptide identification in PMF and MS/MS approaches, we examined the m/z values of the 993 species‐specific peptides found by MS/MS. We found 35 peptide pairs from two different species that fall within a mass difference of less than 20 ppm, and 43 peptide pairs for a mass difference of less than 25 ppm. As the rule of thumb is that X ppm mass tolerance rules out (100 – X)% of peptides^[^ ^50^ ^]^ with identical nominal mass, a ≤20 ppm mass tolerance is usually recommended for differentiation of such peptides. Thus, the 35 pairs ambiguous within 20 ppm were excluded from the list of sequence‐specific peptides. The resultant number of species‐specific peptides identified in different species ranged from 6 (in 9 individual grey seals) to 218 (in 3 individual sperm whales).

Since the PMF approach relies on a combination of at least nine peptide markers, rather than exclusively species‐specific peptides, for reliable identification,^[^ ^51^ ^]^ grey seals may not be reliably identified using PMF due to the limited number of detectable markers for this species.

## 3 Discussion

Here, we create the first installment of an expandable KISSE that can be exploited for the identification of species based on the MS/MS analysis of bone samples using our SIP‐MS model. By analyzing bone samples from 8 different species, we determined that a reliable SSE cannot be built based on sequence‐specific peptides alone. Therefore, we generated a peptide abundance model based on a subset of all quantified peptides.

Although our current SIP‐MS model performed perfectly in identifying species for the tested samples, its performance is constrained by the limited number of species included in the database. With only 8 species represented, the model's predictions are necessarily restricted to this narrow group, while real‐world samples could originate from any taxon. Nevertheless, adding more species, ideally with at least three individuals each, helps in confidently identifying species‐specific peptides, which in turn improves the model's classification accuracy.

During model development, we observed fluctuations in species identification probabilities when splitting the training and test datasets. Species represented by more individuals, such as Steller's sea cow and grey seal (with 9 individuals each), consistently achieved high prediction scores (average of 100%) for the most probable identification. In contrast, species with fewer individuals yielded lower average prediction scores (≈92%). This underlines how greater within‐species sampling improves the model's confidence and reduces misclassification risks.

As for the number of species required to make the model more broadly reliable, this largely depends on phylogenetic distance. For example, distinguishing between whales and seals is relatively straightforward due to their evolutionary divergence. However, distinguishing closely related taxa—such as grey seal, harbor seal, and elephant seal—remains more difficult with the current dataset. At present, we cannot define a precise number of species needed to reach robust performance across taxonomic levels (e.g., genus or family), but increased taxonomic breadth and depth are clearly necessary.

In terms of timeline, we are actively analyzing bone samples from additional species, and expansion is ongoing. However, scaling up meaningfully would require community involvement, either through direct data sharing or systematic acquisition of MS/MS data on known bone samples from a broader array of species. Notably, we recently analyzed two new samples (swan and grey seal), which were not part of the original training set, and the model successfully identified them as swan and grey seal, respectively, demonstrating its extendibility and potential for growth.

We also emphasize the limitation posed by the lack of annotated proteins for many species in public databases such as SwissProt. As of November 2024, there are only 76 reviewed proteins available across all species in our study, and none are collagenous proteins. For example, the harbor seal has 33 annotated proteins, while species such as the whooper swan and Steller's sea cow have none. This presents a substantial obstacle, especially since collagen is among the most abundant and informative proteins in bone. Interestingly, the peregrine falcon and whooper swan both yielded high numbers of species‐specific peptides (182 and 151, respectively), but many of these (91 in falcon and 75 in swan) were annotated as chicken (Gallus gallus) due to the absence of species‐specific references. These results highlight both the potential for misidentification and the need to construct databases from empirical bone proteomes rather than relying solely on annotated sequences.

We also acknowledge that data from archaeological and paleontological contexts typically contain more post‐translational modifications (PTMs) and exhibit greater peptide fragmentation due to degradation. These factors reduce the number and quality of usable peptides, which may compromise model performance if not properly accounted for. While we have not yet tested the model on archaeological samples, we agree that this is a critical next step. Unfortunately, we were unable to find publicly available MS/MS datasets on PRIDE that included both our target species and collagen‐based data, further justifying the need to build dedicated SSEs for underrepresented taxa. The lack of such datasets underscores the value of our initiative, which aims to provide precisely this type of reference.

## 4 Conclusion

The presented SIP‐MS model is the very first step toward a universal SSE. It is also a proof‐of‐principle that even a small sample size (as low as three individuals) can yield reliable species identification in a field where the number of annotated proteins is limited for many species. We would like to emphasize that both “species‐specific peptides” and “informative peptides” are context‐specific to the species database used in KISSE. Any use of these peptides to indicate species specificity in other studies should be approached with caution and supported by thorough examination. The approach described here would greatly benefit from including more collagen‐like proteins in the sequence databases.

## 5 Experimental Section

### Sample Collection

Bone and tooth pieces were obtained from the National Museums Scotland, Edinburgh; A.N. Severtsov Institute of Ecology and Evolution, Moscow, Russia; the Swedish Museum of Natural History, Stockholm, and Stockholm University, both in Sweden. The samples from the Edinburgh and Stockholm museums were all modern, while the seacow and grey whale samples from the Severtsov Institute were between 740 and 4880 years old based on radiocarbon dating. For more details on individual samples, see Table S1 (Supporting Information).

### Collagen Extraction and Proteomics Sample Preparation

Collagen extraction from bones and teeth was carried out using Brown^[^ ^46^ ^]^ protocol. At first, each sample was carefully cleaned and washed with deionized water and air‐dried. Samples were ground to powder using either a dental drill or by a mortar and pestle. ≈50 mg of bone powder from each individual sample was transferred to a glass funnel with an embedded P3 filter (Labglass AB) and glass fiber prefilters (0.7 µm pore size, Merck Millipore) on the top. After the addition of ≈15 mL of 0.25 M HCl (Merck Millipore) to each glass funnel, samples were incubated at room temperature (RT) for 72 h to remove calcium from the collagen matrix (demineralization/decalcification). At the end of incubation time, HCl was drained from the bottom of the funnel by vacuum, and samples were washed with deionized water twice. To denature the collagenous proteins, 10 mL 0.01 m HCl was added to the glass funnel which was incubated at 58 °C for 16 h. After the incubation, the filtrate solution, obtained from the bottom of the glass funnel using a water‐jet pump and vacuum chamber, was transferred to Amicon 15 Ultra‐filtration 30 kDa centrifugal filter (Merck Millipore) and centrifuged at 4 °C for 20 min at 5000 ×g to remove the contaminants and excess of solvent. The solution remaining on top of the filter was then transferred to a 2 mL Eppendorf tube and kept in the freezer at ‐20 °C before being lyophilized to obtain sponge‐looking dried collagen. ≈200 µg of each collagen sample was placed at the bottom of each 1.5 mL Eppendorf tube, and 300 µL of ammonium bicarbonate buffer (50 mm in deionized water with pH = 8, Sigma Aldrich) was added to each tube. Samples were vortexed and then incubated at 70 °C for 3 h to solubilize collagen. Following centrifugation at 14,000 ×g for 10 min, the supernatants were collected. Protein concentration was measured using Bicinchoninic acid assay (BCA, Thermo Fisher Scientific). Each sample was prepared in three replicates, with 20 µg of protein per replicate, and transferred to fresh Eppendorf tubes, and sequence‐grade trypsin (Promega) was added to an enzyme: protein ratio of 1: 50. The samples were incubated overnight at 37 °C in a heat block while mixing at 300 rpm. The next day, formic acid (FA) (98%, Sigma–Aldrich) was added to 5% (v:v, pH in the range of 1 to 3) into each sample to quench the digestion process. Samples were desalted using either C‐18 HyperSep™ Filter Plates (Thermo Fisher Scientific) or Sep‐Pak Vac C‐18 column (Waters) and dried using a Speedvac. Samples were stored at −20 °C before analysis.

### LC‐MS/MS Setting and Data Acquisition

Orbitrap Fusion or Lumos mass spectrometers with ETD MS/MS capability equipped with an EASY‐Spray source were used online with an UltiMate 3000 RSLC nanoUPLC system (all ‐ Thermo Scientific). The dried samples were dissolved in Buffer A (acetonitrile 2%, FA 0.1%, in water, all from Thermo Fisher Scientific) to a 0.5 µg/µL concentration of the tryptic peptides. The peptide mixtures were preconcentrated before injection using a PepMap C18 nano trap column (length ‐ 2 cm; inner diameter ‐ 75 µm; particle size ‐ 3 µm; pore size ‐ 100 Å; Thermo Fisher Scientific) at a flow rate of 3 µL/min for 5 min. Peptide separation was performed using an EASY‐Spray C18 reversed‐phase nano‐LC column (Acclaim PepMap RSLC; length, 50 cm; inner diameter, 75 µm; particle size, 2 µm; pore size, 100 Å; Thermo Fisher Scientific) at 55 °C and a flow rate of 300 nL/min. Peptides were separated using a binary solvent system consisting of 0.1% (v/v) FA, 2% (v/v) acetonitrile in water (solvent A), and 2% water (v/v), 0.1% (v/v) formic acid in acetonitrile (solvent B). The elution gradient was from 4% B to 15% B for 50 min, to 35% B in 10 min, to 95% B in 3 min, staying at 95% B for 7 min, and then decreased to 4% B in 0.5 min and then staying at 4% B for 9.5 min. The mass spectrometry data were acquired in the data‐dependent acquisition mode with selection of top 5 precursor ion. In acquiring survey mass spectra, detection was in the orbitrap analyzer with 120,000 resolution in the m/z range 375–1500. The maximum injection time was 50 ms, and the Automatic Gain Control (AGC) target was 1e6. MS/MS events comprised two different activation type events, ETD and HCD. In the ETD MS/MS event, the ETD reagent target was 1e6 with the supplemental activation collision energy at 30%. In the HCD MS/MS event, the AGC target was set to 2e5 and the HCD energy at 30%. In both types of MS/MS events the quadrupole filter performed ion isolation with a window of 1.4 m/z, the m/z detection range was set to auto mode and the orbitrap nominal resolution was 50,000. All mass spectra were recorded in profile mode.

### Collagen Peptide Identification

The MS/MS data were searched against the collagen and extracellular matrix protein sequences in the reviewed UniProt/SwissProt database in PEAKS Studio^[^ ^49^ ^]^ software version 10.6 (Bioinformatics Solution) using mixed MS/MS activation type (HCD and ETD) and the default setting for all parameters (10 ppm mass error, etc.). Hydroxylation of proline and lysine, deamidation of asparagine and glutamine, and oxidation of methionine were added as variable modifications. The search of MS/MS data against the database was performed with PEAKS DB (with 3 allowed of Post‐Transitional Modifications or PTMs per peptide), PEAKS PTM (with an unlimited number of PTMs), and PEAKS Spider (an algorithm that was specially designed to detect peptide mutations and perform cross‐species homology search) modules. FDR threshold of 5% was applied to filter the list of identified peptides.

### Peptide Quantification

Individual peptide abundances, quantified by measuring the area of the extracted ion chromatogram of the molecular ions from PEAKS Studio output, were first normalized by the total peptide abundance in the corresponding sample and then log10 transformed.

### Building the Species Identification and Prediction by Mass Spectrometry (SIP‐MS) model

After deciding that at least 3 or more individual samples were needed for each species, the species list was reduced down to 8 groups (Cuvier's beaked whale, sperm whale, grey whale, grey seal, seal, Steller's sea cow, peregrine falcon, and whooper swan). In total, 26,581 collagen peptides were identified and quantified for these species.

Three parameters were determined for each peptide as follows:

1. Specificity: 1/N, where N was the number of species that the given peptide was found in.
2. Sensitivity: Number of individuals from the same species in that a given peptide was found divided by the total number of individual samples for the same species.
3. Exclusivity: Square of the total normalized abundance of a given peptide across all samples of the same species divided by the total normalized peptide abundance across all samples used for model building.

A subset of 2,469 peptides, here referred to as “informative peptides,” was selected. The subset comprised 993 species‐specific peptides (those with Specificity and Sensitivity equal to 1) as well as 1476 peptides quantified in at least 25% of all the individual samples with a normalized abundance variation between the species of 3% or higher.

In building the SIP‐MS model, a correlation matrix was 1) first calculated for each sample between the abundances of the informative peptides in the sample vs. those in all the other samples. Then these correlations were averaged for all samples from the same species (giving an “averaged correlation” value). A two‐sided Student t‐test with unpaired and unequal variance between each group was then performed to obtain a p‐value that subsequently could be used to obtain a final prediction score. Then 2) in a second step the selection for each species of the most species‐specific peptides as a subset of those having Specificity and Sensitivity values equal to 1 was obtained. Since the lowest number (11) of species‐specific peptides was found for the grey seal, for each species, the top 11 peptides were chosen after sorting species‐specific peptides by the highest Exclusivity.

A random forest model (by “randomForest”^[^ ^52^ ^]^ package in R version 4.1.0^[^ ^53^ ^]^) was then built using the abundances of the selected 88 species‐specific peptides (i.e. 8X11 peptides). The model provides the 88 abundances of the corresponding peptides and will give the probabilities (Probability_i_) of a data set to belong to any of the 8 species in the database. In order to deal with missing values (NA values), instead of a constant value, the missing values were replaced by a random numbers X from the range:

(1) $$\begin{array}{c} \left(\frac{\min\left(Normalized\;Abundance\right)}{500000}<X<\frac{\min\left(Normalized\;Abundance\right)}{5000}\right) \end{array}$$

This was a necessary step to ensure that the species identification would be based on the presence of peptides rather than their absence. This correction was important, since in the early attempts for creating the model, peptide abundances were imputed with a small constant value, which contributed prominently to erroneous species identification.

The random forest model was trained using approximately two‐thirds of the individuals from each species and tested against the remaining one‐third of the samples to ensure perfect identification.

For a given sample, the prediction score was finally calculated for each species in the database, reflecting the probability, according to the model, of the sample to belong to that species. The Prediction Score_i_ (where *i was* the species index, running currently from 1 to 8) was given by the formula:

(2) $$Prediction\;Score_{i}=\frac{Probability_{i}\times Averaged\;Correlation_{i}\;}{\sum_{i}Probability_{i}\times Averaged\;Correlation_{\;i}}$$

Similarly, the average correlation (Averaged Correlation_i_) was determined from the correlation matrix. Here, the informative peptide abundances for the sample were compared with all individuals in the database, and the correlations were averaged by species.

To identify species, the SIP‐MS model requires as input a table with peptide sequences and normalized abundances that needs to be submitted through the graphical user interface. Detailed instructions on how to upload the table were provided on the SIP‐MS GitHub page (https://github.com/hassanakthv/SIP‐MS) and on the KISSE online tool (https://kisse.serve.scilifelab.se/app/kisse). The SIP‐MS examines if the informative peptides from the predictive model and selected species‐specific peptides from the random forest were present in the submitted table and identifies the most likely species. The KISSE then assesses the statistical certainty of species identification.

### Expansion of the SIP‐MS Model

The SIP‐MS model was designed to be expandable, but certain criteria must be met for the submission to the library of new species. Instructions on how and where to submit a new species to the KISSE were provided at the SIP‐MS GitHub page (https://github.com/hassanakthv/SIP‐MS), as well as the online tool KISSE (https://kisse.serve.scilifelab.se/app/kisse). Briefly, each sample should be well‐documented, preferably with an ID from a museum collection, including information about the bone element, the animal age, sex, place of origin, and other relevant details. For each new species, at least three independent individuals should be available. The added peptide dataset should contain quantified peptide abundances from all individuals. Upon submission, the software verifies that the averaged correlation of the peptide abundances between each individual and the rest of the individuals from the same species was higher than 0.7. Currently, KISSE supports the peptide naming format used by PEAKS Studio. Therefore, peptide names and PTMs representation from other software, such as MaxQuant or FragPipe, must be manually converted to the PEAKS Studio format.

## Conflict of Interest

The authors declare no conflict of interest.

## Supporting information



Supporting Information

Supporting Information

## Data Availability

The mass spectrometry proteomics data have been deposited to the ProteomeXchange Consortium via the PRIDE^[^ ^54^ ^]^ partner repository with the dataset identifier PXD060919. The KISSE GUI source code was available on GitHub (https://github.com/hassanakthv/SIPMS).
